# Small Unruptured Intracranial Aneurysms Can Be Effectively Treated With Flow-Diverting Devices

**DOI:** 10.3389/fneur.2022.913653

**Published:** 2022-05-30

**Authors:** Li Li, Bu-Lang Gao, Qiu-Ji Shao, Guang-Lin Zhang, Zi-Liang Wang, Tian-Xiao Li, Liang-Fu Zhu

**Affiliations:** Henan Provincial People's Hospital, Zhengzhou University, Zhengzhou, China

**Keywords:** flow diverter, intracranial aneurysms, unruptured, small, complications

## Abstract

**Purpose:**

To investigate the effect and safety of flow diverters in the management of small (<10 mm in diameter) unruptured intracranial aneurysms.

**Materials and Methods:**

One hundred and ten patients with 145 small intracranial aneurysms treated with flow diverters were retrospectively enrolled. The clinical, endovascular, and follow-up data were analyzed.

**Results:**

One hundred twenty-one flow diverters were deployed for the treatment of 145 small intracranial aneurysms in 110 patients, and the stenting success rate was 99.1%. In 133 (91.7%) aneurysms, only flow-diverting devices were deployed, and in the rest 12 (8.3%) of aneurysms, coils were used to loosely pack the aneurysm after deployment of a flow-diverting device. Five patients (4.5%) experienced ischemic complications, but no hemorrhagic complications were occurred. All patients had clinical follow-up 6–18 (median 12) after the procedure, with the modified Rankin scale score (mRS) 0 in 101 patients, 1 in four patients, 2 in three patients, 4 in one patient, and 5 in one patient. Digital subtraction angiography was performed at follow-up in 90 (81.8%) patients with 118 (81.4%) aneurysms 6–18 months (median 12) after the procedure, with the Raymond grade I in 90 (76.2%) aneurysms and Raymond grade III in 28 (23.7%). Eighteen patients with 22 partially occluded aneurysms at the first angiographic follow-up experienced the second digital subtraction angiography 12–36 months (median 26) after the procedure, and 21 (95.5%) aneurysms were completely occluded. Two patients had asymptomatic in-stent stenosis.

**Conclusion:**

Treatment of small unruptured intracranial aneurysms with flow diverters can be performed safely and effectively with satisfactory outcomes.

## Introduction

Ever since the approval of flow diversion by the Food and Drug Administration for the treatment of intracranial aneurysms, flow-diverting devices have been increasingly used in the treatment of intracranial aneurysms, especially large and giant aneurysms, which are associated with worse outcomes than small ones (1–8). The indications of treatment for the flow-diverting devices are aneurysms with a maximal diameter of over 10 mm that include large and giant aneurysms and a wide neck with a width of over 4 mm (1, 9) and for aneurysms <10 mm with a narrow neck, traditional stent-assisted coiling embolization is comparatively better. Nonetheless, the management of small intracranial aneurysms remains controversial, with difficulties frequently reported in the literature in both endovascular embolization and surgical clipping, as well as a high crossover rate (up to 18%) from endovascular embolization to surgical clipping (10–12). Favorable clinical and angiographic outcomes of endovascular embolization of small ruptured aneurysms have been reported recently in a study with a long-term follow-up of 5 years (13), which may indicate that experience accumulation may lead to good outcomes. With the accumulation of clinical experience in using the flow-diverting devices for intracranial aneurysms, the indication of flow diversion has been greatly expanded from large and giant unruptured aneurysms to ruptured, blister, and dissecting aneurysms as off-label use (14). This is because flow-diverting devices are different from conventional regular arterial stents in that they have a higher metal coverage surface to divert blood flow away from the aneurysm, promote flow stasis and thrombosis within the aneurysm cavity, and remodel the parent artery for aneurysm regression (14, 15). These advantages can be used to treat complex, large, and giant intracranial aneurysms, which are hard for traditional endovascular or surgical approaches, resulting in a low complication rate and a low recurrence rate (14, 16–21). For small aneurysms <10 mm in diameter (22, 23), the flow-diverting devices also have some specific advantages, such as simple operation and low intraprocedural aneurysm rupture rates, and thus can be used in the treatment of small aneurysms. It was hypothesized that flow-diverting devices could be safely and effectively used in the treatment of small intracranial aneurysms. This study was consequently performed to investigate the effect and safety of flow-diverting devices in the treatment of small intracranial aneurysms.

## Materials and Methods

### Subjects

This retrospective study was approved by the ethics committee of our hospital, and all patients or their family members had given the signed informed consent to participate. From March 2014 to April 2019, patients with small unruptured intracranial aneurysms treated with flow-diverting devices in our hospital were enrolled. The inclusion criteria were consecutive patients with small (<10 mm) unruptured intracranial aneurysms, which were treated with flow-diverting devices (Pipeline Embolization Device, Medtronic, Irvine, CA, USA, and Tubridge, MicroPort Medical Company, Shanghai, China) that include saccular, dissecting, or fusiform aneurysms. The exclusion criteria were patients with larger (>10 mm) aneurysms, ruptured aneurysms, and aneurysms, which had been treated previously using surgical clipping or endovascular embolization.

### Endovascular Procedure and Medication

Three to 5 days before the endovascular procedure, dual antiplatelet therapy was administered for all patients with clopidogrel (75 mg/d) and aspirin (100 mg/d) (24). The endovascular procedure was conducted under general anesthesia and heparinization. Percutaneous access was obtained using femoral artery puncture, and a microcatheter was navigated through the guiding catheter to the aneurysm. An appropriate flow-diverting device was selected and sent to the right location for deployment. In aneurysms with an aneurysm neck > 7 mm, an irregular dome, or a daughter sac, coils were used to embolize the aneurysm. For patients with the device being opened poorly, long operation time in the procedure, and suspected thrombosis at the aneurysm neck, Tirofiban was administered intravenously after stent deployment (25), with the beginning injection dose of 5 μg/kg injected within 3 min followed by instillation in the dose of 0.05 μg/kg^−1^/min^−1^, which was 1/2 of the conventional dose. After the endovascular procedure, Tirofiban was continually administered for 24–36 h, and aspirin (100 mg/d) and clopidogrel (75 mg/d) were continued in all patients for 3 months followed by long-term use of aspirin (100 mg/d) alone.

Clinical and angiographic follow-up was scheduled in all patients, and the treatment effect of the flow-diverting device was evaluated 6 months after the procedure using the Raymond grading system (26), with the Raymond grade I as complete obliteration of aneurysm, grade II as a residual neck, and grade III as any opacification of the aneurysm sac or residual aneurysm. The clinical prognosis was assessed with the modified Rankin scale (mRS) scores.

### Statistical Analysis

Statistical analysis was performed with the SPSS software version 19.0 (IBM, Chicago, IL, USA). Continuous data in normal distribution were presented as mean ± standard deviation (SD). Enumeration data were presented as frequency and percentages.

## Results

One hundred and ten patients with 145 aneurysms who met the inclusion criteria were enrolled that include 77 (70%) male and 33 (30%) female patients with an age range of 35–78 years (mean 53.7 ± 18.3; Table 1). Clinical symptoms included headache or dizziness in 56 (50.9%) patients and ischemic cerebral diseases in 19 (17.3%) patients. The rest 35 (31.8%) patients were incidentally found. Aneurysm location involved the internal carotid artery (ICA) C4–C7 segments in 131 (90.3%) aneurysms, ICA C2 segment in two (1.4%), V4 segment of the vertebral artery in nine (6.2%), middle cerebral artery M1 segment in one (0.7%), and M2 segment in two (1.4%). Among 110 patients, 77 (70%) patients had one aneurysm each, 22 (20%) had two aneurysms each, and eight (5.5%) had three aneurysms each.

**Table 1:** Demography, clinical data, and endovascular treatment.

**Variables**		**Data**
Patients	F/M	33/77
	Age (y)	35–78 (53.7 ± 18.3)
Symptoms (*n*, %)	Headache or dizziness	56 (50.9%)
	Ischemic symptoms	19 (17.3%)
	Incidentally found	35 (31.8%)
Aneurysm location (*n*, %)	ICA C4-C7	131 (90.3%)
	ICA C2	2 (1.4%)
	Vertebral V4 segment	9 (6.2%)
	MCA M1 segment	1 (0.7%)
	MCA M2 segment	2 (1.4%)
No. of aneurysms (*n*, %)	Patients with one aneurysm each	77 (70%)
	Patients with 2 aneurysms each	22 (20%)
	Patients with 3 aneurysms each	8 (5.5%)
Stenting procedures	No. of flow diverters deployed	121
	Diverters only	133 (91.7%)
	Diverters and coiling combined	12 (8.3%)
	Success rate of procedure	99.1% (109/110)
Periprocedural complications	Ischemic	5 (4.5%)
	Hemorrhagic	0
Clinical follow-up	Duration (m)	6–18 (median 12)
	mRS 0 (*n*, %)	101 (91.8%)
	mRS 1 (*n*, %)	4 (3.6%)
	mRS 2 (*n*, %)	3 (2.7%)
	mRS 4 (*n*, %)	1 (0.9%)
	mRS 5 (*n*, %)	1 (0.9%)
Angiographic follow-up at 6–18 months	No. of patients (*n*, %)	90 (81.8%)
	No. of aneurysms (*n*, %)	118 (81.4%)
	Raymond grade I	90 (76.2%)
	Raymond grade III	28 (23.7%)
Angiographic follow-up at 12–36 months (median 26)	No. of patients (*n*, %)	18 (16.4%)
	No. of aneurysms (*n*, %)	22 (15.2%)
	Raymond grade I	21 (95.5%)
	Asymptomatic instent stenosis (*n*)	2 (9.1%)

*ICA, internal carotid artery; MCA, middle cerebral artery; mRS, modified Rankin scale score*.

**Figure 1:**
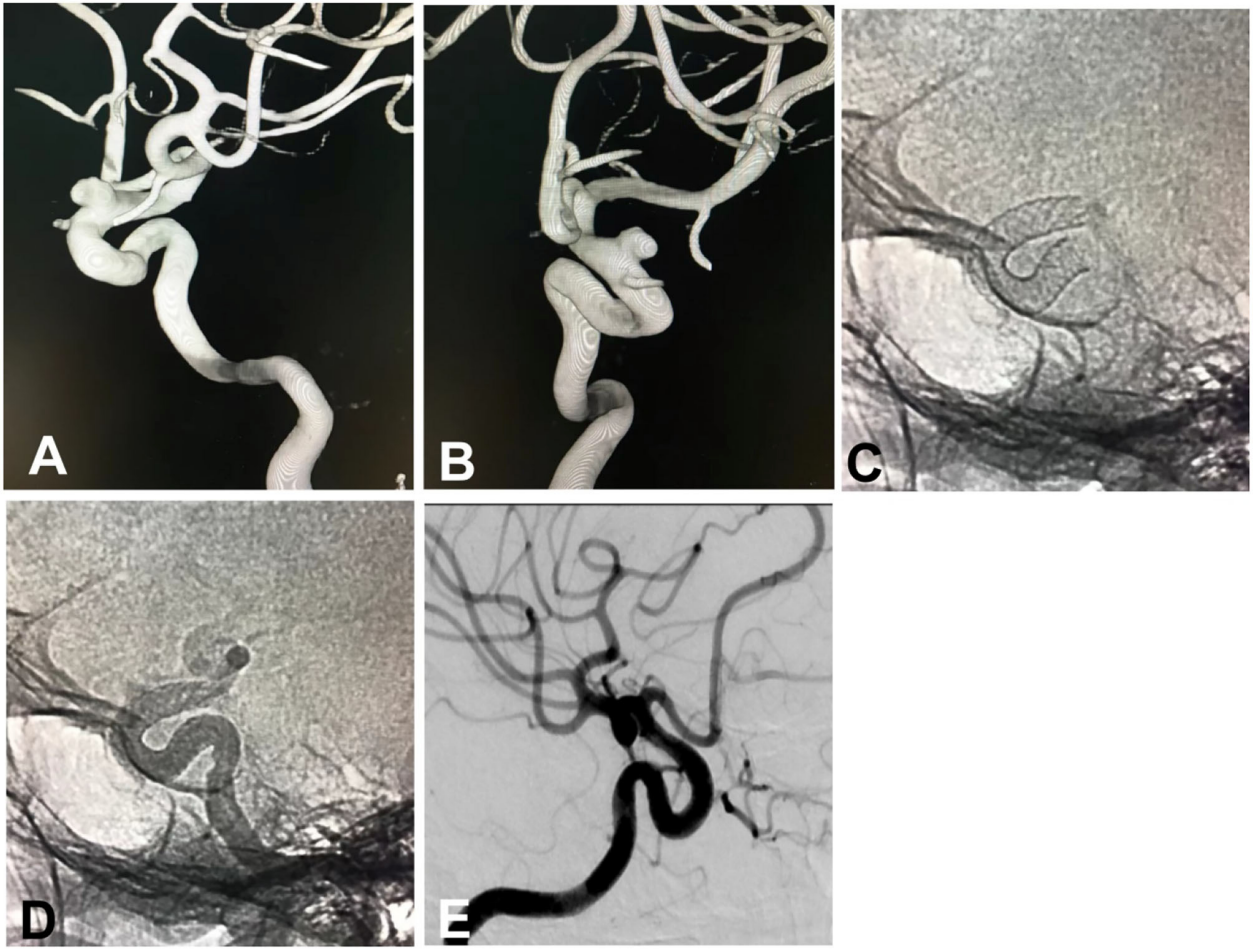
A small intracranial aneurysm in a 53-year-old woman with dizziness was treated with the deployment of a Pipeline embolization device. **(A,B)** The small aneurysm was located at the sixth (ophthalmic) segment of the left internal carotid artery, measuring 5.1 × 3.0 mm in the sac with a 4-mm neck. **(A)** Lateral position, **(B)** Oblique position. **(C)** Immediately after deployment of a Pipeline device of 4.25 × 20 mm, the stent was shown to have a good opening on angiography. **(D)** Angiography immediately following the deployment of the stent, the parent artery was shown to be patent with good wall adherence of the stent. **(E)** Follow-up angiography 8 months later revealed patent parent artery and complete occlusion of the aneurysm.

In the endovascular procedure, 121 flow-diverting devices were deployed to treat 145 aneurysms (Figure 1) that include 20 (16.53%) Tubridge and 101 (83.47%) Pipeline devices. In 133 (91.7%) aneurysms, only flow-diverting devices were implanted, and in the rest of 12 (8.3%) aneurysms, coils were inserted into the aneurysm sac for loose packing after placement of the flow-diverting device. In one patient with a 4-mm aneurysm at the tortuous paraclinoid segment of ICA, the distal end of the Tubridge device (4.5 × 30 mm) was herniated into the aneurysm cavity when the micro-guidewire was withdrawn, and repeated attempts did not succeed in correct deployment of the Tubridge stent, resulting in failure of stenting. All the other patients had successful stent deployment, with a success rate of stenting of 99.1% (109/110). In 17 devices, good opening and wall adherence were obtained with balloon expansion or micro-guidewire “massage” after stent deployment.

Five peri-procedural ischemic complications occurred, i.e., one patient who was treated with a Pipeline device combined with coiling and four treated with the deployment of a Pipeline device only, resulting in a complication rate of 4.5%. No hemorrhagic complications took place in this cohort. In the case with the distal stent end herniating in the aneurysm cavity, the parent artery was occluded, and the anterior cerebral artery had good compensation, resulting in an mRS of 2 at a 3-month follow-up. In one case with poor stent adherence to the arterial wall, cerebral infarction had occurred in the area supplied by the choroidal artery and anterior cerebral artery covered by the stent, resulting in an mRS score of 4 at follow-up evaluation. In one case with in-stent thrombosis leading to occlusion of the middle cerebral artery, the mRS was 5 at follow-up. In one case with an atherosclerotic plaque at the parent artery near the aneurysm neck leading to slight stenosis of the parent artery, in-stent thrombosis occurred 4 h after the procedure, and acute endovascular thrombectomy was performed, resulting in recanalization and good recovery of the patient. Complete occlusion of the aneurysm and an mRS of 0 were present at the 3-month follow-up. In one case with cerebral ischemic symptoms (hemiplegia), intravenous pumping of Tirofiban resulted in good recovery.

All patients had clinical follow-up 6–18 (median 12) after the procedure, with the mRS 0 in 101 (91.8%) patients, 1 in four (3.6%) patients, 2 in three (2.7%) patients, 4 in one (0.9%) patient, and 5 in one (0.9%) patient. Digital subtraction angiography was performed at follow-up in 90 (81.8%) patients with 118 (81.4%) aneurysms 6–18 months (median 12) after the procedure, with the Raymond grade I in 90 (76.2%) aneurysms and Raymond grade III in 28 (23.7%). Eighteen patients with 22 partially occluded aneurysms at the first angiographic follow-up experienced the second digital subtraction angiography 12–36 months (median 26) after the procedure, and 21 (95.5% or 21/22) aneurysms were completely occluded (Raymond grade I, Figure 1). Two patients had asymptomatic in-stent stenosis.

## Discussion

In this study, the safety and effect of flow diverters in treating small unruptured intracranial aneurysms were investigated, and it was found that treatment of small unruptured intracranial aneurysms with flow diverters could be performed safely and effectively with satisfactory outcomes.

Small intracranial aneurysms refer to a aneurysms with the maximal diameter <10 mm regardless of the aneurysm nature, such as saccular, dissecting, or fusiform, accounting for a large proportion of cerebral aneurysms (22, 23, 27). These small aneurysms may be irregular, with daughter sacs, in the posterior circulation, and should be treated actively to prevent possible rupture even though they are not the conventional indications for use of flow diverters. Traditionally, stent-assisted coiling has achieved good clinical and imaging outcomes in the treatment of small (<10 mm) unruptured intracranial aneurysms (22). However, endovascular treatment of intracranial aneurysms that include small unruptured ones still faces great challenges, such as incomplete occlusion, recurrence of wide-necked aneurysms, difficult access or unstable placement of embolization catheters due to anatomical characteristics of aneurysms or poor remodeling, intraprocedural rupture during the process of dense embolization, and difficulties and complex management of multiple tandem aneurysms. Thus, it is natural to use flow diverters for the treatment of these kinds of aneurysms (28). The advantages of using flow diverters for these aneurysms included simplified operation with no need to use an embolization catheter into the aneurysm sac for coiling, decreased recurrence or retreatment rate in wide-necked and complex aneurysms with increased long-term effects, and loose packing in some aneurysms with no or decreased risk of aneurysm rupture. However, in conventional stent-assisted coiling, the embolization outcome may be affected by stent types, size of the first coil, proper shaping of the microcatheter, packing of the last coil, and dense packing.

Because of the advantages of flow-diverting devices, the embolization operation with flow-diverting devices is not so difficult, and the rate of peri-procedural complications is decreased. No patients experienced hemorrhagic complications, and the ischemic complication rate was only 4.5% in our study. The ischemic complication rate had been reported to be 2.7 (19) and 8.7% (28) in the use of flow-diverting devices for the treatment of intracranial aneurysms and 4.6–11.2% in traditional stent-assisted coiling of intracranial aneurysms (22, 29), similar to ours. In a meta-analysis of 41 studies that involved 2,614 patients with aneurysms <10 mm treated with flow diverters, the complication rate was reported to be 7.8% (95% CI 4.8–11.4%) (30), and another meta-analysis that investigated the safety and efficiency of flow diverters in treating small aneurysms (<10 mm) also reported procedural-related neurological mortality of 0.87% and morbidity of 5.22%. These complication rates in these meta-analyses were similar to the above complication rates of intracranial aneurysms treated with either flow diverters or stent-assisted coiling. Many reasons may contribute to the occurrence of ischemic complications, such as inexperience, poor adherence or insufficient opening of the stent, insufficiency of antiplatelet therapy, and adjunctive coiling. In our study, the five ischemic complications may probably be associated with the early inexperience in using the flow diverters and possible stenosis of the parent artery. To decrease the ischemic complications, the following aspects should be paid attention to. Because poor wall adherence is an independent risk factor for ischemic complications (31), the flow-diverting device should be deployed to have good wall adherence. With experience accumulation in the process of learning, the flow-diverting device can be deployed with good wall adherence, and the technical complication rate related to wall adherence can be significantly decreased. Moreover, adequate antiplatelet therapy should be administered in the peri-procedural period to prevent possible ischemic complications. In our study, a small dose of Tirofiban was used 24–48 h after deployment of the device in patients with good thromboelastogram, which can significantly decrease ischemic complications without increasing the risk of rupture of intracranial aneurysms based on our experience (25, 32). In patients with parent artery stenosis >50%, balloon expansion should be performed in advance to relieve the stenosis before deployment of the flow-diverting device so as to obtain good wall adherence after deployment. The use of a microcatheter for “massaging” the flow diverter or a balloon to expand the flow diverter can effectively increase the rate of good wall adherence.

In our study, the aneurysm complete occlusion rate (Raymond grade I) was 76.2% at the first angiographic follow-up 6–18 months after the procedure, but 95.5% at the second angiographic follow-up 12–36 months (median 26) after the procedure, similar to those reported by other researchers in the treatment of intracranial aneurysms using traditional stent-assisted coiling (33) or flow diverters (34). Complete occlusion of the aneurysms may depend on several factors. Firstly, the long-term outcome of aneurysm occlusion primarily relies on neointima to completely cover the aneurysmal neck, which may require a period of 20–24 wk based on animal experiments (35). A short period of time between 6 and 18 months may not be sufficient for the neointima to cover the aneurysm neck for complete occlusion. Moreover, aneurysm occlusion outcome may also be affected by the stent adherence, metal coverage, and parent artery tortuosity. Aneurysm complete occlusion rate after treatment with flow diverters has an apparent time dependence, with the complete occlusion rate of 73.6% at 6-month follow-up but 95.2% at 5 years after endovascular treatment (34), similar to the outcomes in our study. An adjusted complete occlusion rate of 74.9% (95% CI of 69.6–79.8%) of aneurysms <10 mm has been reported at 12 months after treatment with flow diverters in a meta-analysis (30). A complete occlusion rate of 84.23% (95% CI 80.34–87.76%) has also been reported in a systematic review and meta-analysis investigating the safety and efficiency of flow diverters in the treatment of small aneurysms <10 mm (27). The use of one or multiple flow diverters may also affect aneurysm complete occlusion rate, with multiple diverters being frequently deployed for large and giant aneurysms and one diverter for small aneurysms. It may thus be more appropriate to define the primary end point of the use of flow diverters in the treatment of aneurysms as the cure rate from 12 to 18 months after treatment.

Metal coverage and mesh size of the stent at the aneurysm neck may be factors significantly affecting the complete occlusion rate of intracranial aneurysms (36), and additional coiling in conjunction with the Pipeline embolization device may effectively increase the complete occlusion rate of intracranial aneurysms, especially for large and giant ones (37). Nonetheless, adjunctive coiling after deployment of a flow diverter may increase the risk of ischemic stroke (38, 39). In our practice, additional coiling was usually performed only in irregular aneurysms > 10 mm with daughter sacs. For small unruptured intracranial aneurysms, it is not necessary to use coils in conjunction with flow diverters for complete aneurysm occlusion so as to avoid increased operation difficulty and risk of complications.

This study had some limitations, such as the retrospective and one-center study nature, no control, no randomization, and Chinese patients enrolled only. Future studies with randomization, control, and multiple centers will have to be performed to resolve these issues for better outcomes.

In conclusion, the use of flow diverters in the treatment of small unruptured intracranial aneurysms may result in good outcomes and fewer peri-procedural complications and may become the preferential choice for small unruptured aneurysms.

## Data Availability Statement

The original contributions presented in the study are included in the article/supplementary material, further inquiries can be directed to the corresponding author/s.

## Ethics Statement

The studies involving human participants were reviewed and approved by Ethics Committee of Henan Provincial People's Hospital. The patients/participants provided their written informed consent to participate in this study.

## Author Contributions

LL and T-XL: study design. LL, B-LG, Q-JS, G-LZ, Z-LW, and L-FZ: data collection. LL, B-LG, and T-XL: data analysis. Z-LW and L-FZ: study supervision. LL: writing of the original version. B-LG: revision of the original version. All authors agree to be accountable for all aspects of the work and approve the final version of the article.

## Funding

This study was supported by the 13th Five-year Plan of China for Research and Development (2016YFC1300702), Henan Province Science and Technology Key Project (182102310658), and Scientific and Technological Project of Henan Province (222102310208).

## Conflict of Interest

The authors declare that the research was conducted in the absence of any commercial or financial relationships that could be construed as a potential conflict of interest.

## Publisher's Note

All claims expressed in this article are solely those of the authors and do not necessarily represent those of their affiliated organizations, or those of the publisher, the editors and the reviewers. Any product that may be evaluated in this article, or claim that may be made by its manufacturer, is not guaranteed or endorsed by the publisher.
